# Characterizing Six Percolation Cases in Flexible Electronic Composites: A Monte Carlo-Based 3D Compressive Percolation Model for Wearable Pressure Sensors

**DOI:** 10.3390/ma18030685

**Published:** 2025-02-04

**Authors:** Sang-Un Kim, Joo-Yong Kim

**Affiliations:** 1 Department of Smart Wearable Engineering, Soongsil University, Seoul 06978, Republic of Korea; tkddnsl0723@naver.com; 2 Department of Materials Science and Engineering, Soongsil University, Seoul 06978, Republic of Korea

**Keywords:** 3D compressive percolation, flexible electronic composites, wearable pressure sensor, Monte Carlo simulation

## Abstract

This study employs a Monte Carlo-based 3D compressive percolation model to systematically analyze the electrical behavior of flexible electronic composites under compressive deformation. By simulating the spatial distribution and connectivity of conductive particles, this study identifies six distinct percolation cases, each describing a unique connectivity evolution under strain. The model reveals that excessive initial connectivity leads to saturation effects, reducing sensitivity, while a high Poisson’s ratio (≥0.3) causes connectivity loss due to shear plane expansion. Notably, asymmetric particle shapes, such as cylinders and rectangles, exhibit superior percolation behavior, forming infinite clusters at lower strain thresholds (~0.4) compared to spherical particles (~0.5). Monte Carlo simulations with 3000 particles validate these findings, showing consistent trends in percolation behavior across different deformation states. By classifying and quantifying these six connectivity scenarios, this research provides a structured framework for optimizing flexible sensor designs, ensuring an optimal balance between conductivity and sensitivity. These findings contribute to advancing flexible electronics, particularly in wearable health monitoring, robotics, and smart textiles.

## 1. Introduction

As nanoparticles with excellent electrical properties such as carbon black [1,2], graphene [3,4], carbon nanotube [5,6], and MXene [7,8,9,10] have been actively researched, these studies have extended to the development of flexible electronic materials through composite fabrication. Flexible electronic composites represent a cutting-edge material trend by integrating conductive particles into flexible substrates, thereby combining the advantages of both components. Unlike conventional rigid substrates used in many electronic devices, these flexible composites exhibit unique electrical properties alongside mechanical flexibility [11,12,13,14].

These materials have found applications in various advanced technological fields, such as wearable sensors, electronic skins, and flexible displays. Wearable sensors have garnered significant attention, leveraging the principle that the material’s deformation and corresponding changes in electrical properties are influenced by human movement or physiological signals [15,16,17,18]. Flexible electronic composites have also been utilized as electrodes for collecting electrical signals, such as electrocardiograms (ECGs) and electromyograms (EMGs), by adhering closely to the skin [19,20]. Additionally, they serve as band-type sensors that monitor respiration by detecting changes in abdominal volume during inhalation and exhalation [21]. Other applications include sensors attached to joints or muscles [22], which measure stretching or bending degrees to recognize physical activities [23]. These examples highlight the extensive potential of flexible electronic composites in diverse applications.

One fundamental mechanism underlying the sensing of such physiological signals is piezo resistivity. When pressure deforms the material, the conductive particles embedded within its structure move closer together or further apart, resulting in measurable changes in the overall resistance of the composite [24,25]. This property is central to the functionality of flexible sensors designed for detecting biomechanical and physiological signals. The behavior of nanoscale conductive particles and the corresponding changes in a material’s electrical properties are effectively described by percolation theory, a foundational framework in this study [26,27]. This theory evaluates the arrangement and interconnectivity of conductive particles embedded within a flexible matrix material, where the matrix primarily determines mechanical properties, and the particles define electrical characteristics [28].

**Figure 1:**
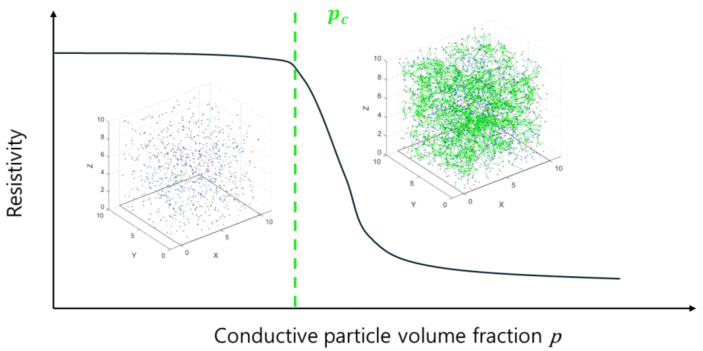
Percolation theory of conductive composites.

As shown in Figure 1, the spatial distribution of conductive particles is quantified using the volume fraction probability *p*, which represents the fraction of the total material volume occupied by the conductive particles. When these particles are positioned sufficiently close to one another, they form conductive paths, collectively referred to as clusters. As the volume fraction *p* increases, the clusters grow in size and begin to coalesce, eventually forming a continuous network that spans from one side of the structure to the opposite side. This large-scale conductive network is known as the infinite cluster. The critical point at which this infinite cluster forms is defined by the critical volume fraction $p_{c}$. At $p_{c}$, the material transitions from an insulating to a conductive state due to the establishment of long-range connectivity. The electrical conductivity of the composite is significantly enhanced by the formation of the infinite cluster, and its strength is determined by the number and arrangement of conductive particles within it. This critical phenomenon is central to understanding and optimizing the electrical properties of conductive composites. The following equation can explain the percolation theory representing electrical resistance.

(1) $$R\left(p\right)=\frac{1}{\sigma (p)}=\frac{1}{{\sigma_{0}(p-p_{c})}^{t}}$$

where the $\sigma \left(p\right)$ is the electrical conductivity at the particle volume fraction p, $\sigma_{0}$ is the proportionality constant, and t is the experimentally determined critical exponent. When $p<p_{c}$, insufficient connectivity results in conductivity approaching zero and resistance tending to infinity; when $p>p_{c}$, conductive pathways form, leading to a sharp increase in conductivity and a decrease in resistance. Near $p\approx p_{c}$, conductivity exhibits nonlinear growth governed by the critical exponent ttt, highlighting strong transition characteristics.

A related study by M. Åkerfeldt et al. used percolation theory to analyze the electrical properties of composites by adding the conductive polymer PEDOT-PSS to a polyurethane (PU) binder. The study showed a significant decrease in surface resistance at around 2 wt% PEDOT-PSS, indicating the formation of a conductive network. Beyond this concentration, the resistance plateaued, highlighting a critical threshold for achieving electrical connectivity [29]. Similarly, the study by Wang et al. demonstrated the occurrence of a percolation zone in mechanical sensing applications using flexible materials. This zone was characterized by a significant decrease in electrical resistance as the content of conductive fillers increased, highlighting the critical role of filler concentration in achieving electrical conductivity [30]. When fabricating flexible composite materials, numerous studies have shown that adjusting the volume fraction of conductive particles within the matrix to exceed the critical volume fraction $p_{c}$ enhances electrical properties. However, for wearable sensors, sensitivity—arguably the most critical performance metric—requires significant changes in electrical properties to detect subtle variations effectively. Therefore, applying percolation theory to analyze and optimize the deformation behavior of flexible electronic composites is crucial for achieving high sensitivity.

To address this, the present study references a Monte Carlo method [28,31,32], leveraging random number generation to simulate complex systems and extract data for probability-based numerical calculations. This approach considers the inherent challenges in controlling the distribution of nanoscale conductive particles within a flexible material matrix. Additionally, a 3D compressive percolation system was developed as an optimization simulation for wearable sensor sensitivity. This system enabled the analysis of electrical property changes under compression, providing insights into trends and behaviors for real-world applications.

## 2. Materials and Methods

### 2.1. Percolation Model

The percolation model used in this study was developed using MATLAB R2023a. The model simulates conductive particles randomly distributed within a defined cubic space, with particle sizes also varying randomly within a specified range. To account for the typical conductive fillers—such as carbon black, CNTs, graphene, and MXenes—the particle shapes were categorized as sphere (0D), cylinder (1D), and rectangular (2D), serving as key variables in the simulation. Connectivity within the percolation model was defined such that particles are considered connected when the distance between them falls below a specific threshold, termed the connectivity length, *ξ*. The relationship between the distance of two particles and the connectivity length determines the network formation, which can be expressed as follows:

(2) $$r_{ij}=\left|r_{i}-r_{j}\right|$$

(3) $$A_{ij}=\left\{\begin{array}{c} 1,\;\;r_{ij}\leq \xi \\ 0,r_{ij}>\xi \end{array}\right.$$

where the $r_{ij}$ represents the distance between particles i and j; $r_{i}$ and $r_{j}$ are their position vectors; and $A_{ij}$ is the adjacency matrix element indicating connection. In addition to the arrangement of conductive particles in Equation (2), an equation that considers the size of the particles can be expressed as follows:

(4) $$A_{ij}=\left\{\begin{array}{c} 1,\;\;r_{ij}-(R_{i}+R_{j})\leq \xi \\ 0,r_{ij}-(R_{i}+R_{j})>\xi \end{array}\right.$$

where the *R* represents the radius of particles i and j. It indicates that a connection can be made if the distance between the centers of particles is within the critical distance minus the size of the particles. The final equations related to the alignment and connectivity of conductive particles, considering three distinct shapes (sphere, cylinder, and rectangular), can be expressed as follows:

(5) $$P_{connect,sphere}\left(r_{ij}\right)=exp\;(-\frac{r_{ij}-(R_{i}+R_{j})}{\xi })$$

(6) $$P_{connect,cylinder}\left(r_{ij},\theta_{i},\theta_{j}\right)=exp\;(-\frac{r_{ij}-\left(R_{i}+R_{j}+L_{i}cos\theta_{i}+L_{j}cos\theta_{j}\right)}{\xi })$$

(7) $$P_{connect,rectangular}\left(r_{ij},\theta_{i},\theta_{j},a_{i},\;a_{j},\;b_{i},\;b_{j}\right)=exp\;(-\frac{r_{ij}-\sqrt{{{(a}_{i}cos\theta_{i}+a_{j}cos\theta_{j})}^{2}+{{(b}_{i}sin\theta_{i}+b_{j}sin\theta_{j})}^{2}}}{\xi }$$

where $L_{i}\;\mathrm{and}\;L_{j}$ are the effective length of cylinder particles i and j; $\theta_{i}\;\mathrm{and}\;\theta_{j}$ are the angular alignments i and j; $a_{i},\;a_{j},\;b_{i},\;{\mathrm{and}\;b}_{j}$ are the length of major axis and minor axis; and $P_{connect,sphere}$, $P_{connect,cylinder}$, and $P_{connect,rectangular}$ are the connection probabilities. These equations describe the connectivity between particles by incorporating their size, alignment, and orientation for each geometry type.

The percolation model, described by Equations (5)–(7), defines the initial connectivity of a conductive network by incorporating the influence of different particle geometries—spherical, cylindrical, and rectangular—on electrical conductivity. In the case of spherical particles, as expressed in Equation (5), connectivity depends solely on the spatial proximity of two particles, meaning that a conductive path is formed if the center-to-center distance between two spheres is within a defined threshold ξ, with connectivity probability $P_{connect,sphere}$ being isotropic due to the uniform geometry of the spheres. However, in cylindrical particles, governed by Equation (6), connectivity is influenced by both spatial proximity and directional alignment, where two cylinders can only form a conductive link if they are not only within the threshold distance ξ but also aligned at favorable angles $\theta_{i}$ and $\theta_{j}$, with their effective lengths $L_{i}$ and $L_{j}$ determining the extent of possible conductive interaction, thereby introducing anisotropic connectivity characteristics. In contrast, rectangular particles, as described in Equation (7), exhibit even more pronounced anisotropic behavior, where connectivity is affected by both the major axis a, which influences long-range connectivity, and the minor axis b, which affects local contact probability, while the orientation angles $\theta_{i}$ and $\theta_{j}$ dictate the likelihood of forming a conductive path, meaning that connectivity is highly dependent on both geometric proportions and spatial positioning.

The electrically connected group of conductive particles is referred to as a cluster. Within a cluster, the free electrons of the conductive particles facilitate charge transport, thereby forming an electrically conductive network. For a composite material to exhibit effective conductivity, it is not sufficient to have finite clusters that are isolated within the material. Instead, as the volume fraction of conductive particles increases, these finite clusters gradually merge, forming a continuous network that spans the entire structure from one side to the other. This ultimately leads to the formation of an infinite cluster, which can also establish connectivity with external electrodes, enabling the material to function as a conductive composite.

The fundamental percolation model in this study was designed to distinguish and analyze various parameters, including the number of conductive particles, particle size, shape, and connectivity length, as well as the definitions of finite and infinite clusters. The model calculates the number of clusters and the number of particles within the infinite cluster, providing insights into the electrical properties of the material. This approach enables a systematic evaluation of how these factors influence the transition from isolated clusters to a fully connected conductive network. This model allows for systematic analysis of how particle shape, size, and distribution influence the percolation threshold and overall network connectivity.

### 2.2. Three-Dimensional Compressive Percolation

From the basic percolation model, this study extends the analysis to model a 3D compressive percolation system, simulating the behavior of flexible electronic composites under compressive deformation when used as pressure sensors. The Poisson’s ratio, which may vary depending on the flexible substrate, was also considered in this model. Instead, the focus was on analyzing the changes in clusters as the initial distribution of conductive particles proportionally shifted along the compression direction.

To incorporate the effects of compression, a new variable, compressive strain, was introduced into the percolation model. This variable accounts for the positional shifts in particles under compression, leading to closer connections between them. Using this modified model, the total number of clusters, the number of infinite clusters, and the number of particles constituting the infinite cluster can be expressed as follows:

(8) $$L_{connection}\left(\varepsilon ,r_{ij}\right)=\frac{N(N-1)}{2}exp\;(-\frac{\left(1-\varepsilon \right)r_{ij,0}}{\xi })$$

(9) $$C\left(\varepsilon ,r_{ij}\right)=\alpha L_{connection}$$

(10) $$n_{i.c}\left(\varepsilon \right)=n_{max}(1-\exp\;\left(-k\left(P_{connection}\left(\varepsilon \right)-P_{c}\right)\right))$$

where *N* is the number of particles, *ε* represents the compressive strain, C is the total number of clusters, α is a proportionality constant, $L_{connection}$ determines the connection probability under compression, $n_{i,c}\left(\varepsilon \right)\;$ is the number of particles in the infinite cluster under compressive strain, and $n_{max}$ is the maximum sensitivity. These equations effectively describe the behavior of particle connectivity and cluster formation under compressive strain, providing a framework to analyze and optimize the performance of flexible electronic composites.

While Equations (5)–(7) define the initial network structure of percolation model under static conditions, Equations (8)–(10) extend this framework by incorporating compressive strain ε to model dynamic changes in connectivity. Equation (8) describes how the total number of clusters *N* evolves as a function of strain, explaining that as compressive deformation increases, more particles transition from isolated states to connected conductive paths; Equation (9) focuses on the formation of the infinite cluster, which represents a fully connected conductive network spanning the material, showing that the connectivity probability $P_{c}$ and the applied strain ε dictate whether a percolating network emerges or collapses; and, finally, Equation (10) defines maximum sensitivity $n_{max}$ by quantifying the strain-dependent growth of electrical conductivity, demonstrating that the relationship between compressive strain, cluster formation, and connectivity probability determines the overall electrical performance of the material. It is possible to analyze how different particle shapes, orientations, and initial distributions influence the formation of conductive pathways and predict how external mechanical deformation affects electrical connectivity, thereby enabling the optimization of flexible electronic composites for high-performance applications.

### 2.3. Monte Carlo Method

In the developed 3D compressive percolation model, the Monte Carlo method was employed to collect data under random conditions, enabling statistical parameter estimation for the model. Among the input variables, factors such as the initial positions, sizes, and orientations of the conductive particles—which are uncontrollable in real flexible electronic composites—were assigned randomly. Meanwhile, controllable factors were carefully regulated. The composite’s dimensions were fixed at a 10 × 10 × 10 cubic structure, and the Poisson’s ratio was varied between 0 and 0.3. The number of conductive particles ranged from 0 to 4500, while their shapes were categorized into spheres, cylinders, and rectangles. The connectivity length was defined as the threshold distance at which particles were considered to be in contact, and the compressive strain was set at 0.8.

The output data included the number of finite clusters, the connections in the infinite cluster, and the maximum number of conductive particles in the infinite cluster. These outputs provided a comprehensive understanding of the structural and electrical characteristics of the flexible electronic composite under compression.

## 3. Results

### Case of 3D Compressive Percolation

**Figure 2:**
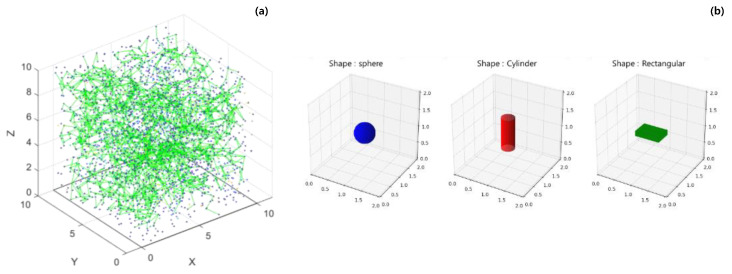
The results of the 3D modeling images: (**a**) 3D compressive percolation model, (**b**) the 3 shape of conductive particles.

Figure 2a shows a characteristic image of the 3D compressive infiltration model generated using the Monte Carlo method. The blue dots represent conductive particles randomly placed throughout space, and the image is scaled down to 1:10 of the size of the placed particles for visualization. The green lines represent connections between these particles. These connections indicate the formation of infinite clusters, and more green lines indicate increased connectivity, reflecting the enhanced electrical conductivity of the material. Figure 2b illustrates the three shapes of conductive particles used in the model: sphere, cylinder, and rectangular. These shapes were selected to study the impact of particle geometry on the percolation behavior and overall conductivity of the composite material.

Table 1 presents the results of the number of connected particles within the infinite cluster under the same compression conditions and within a given particle size range, where particle sizes and spatial distributions were randomly assigned. Despite conducting the Monte Carlo simulation under identical conditions—with 3000 particles and a Poisson’s ratio of 0—the first result yielded an initial connectivity of 0.28, which was higher than the second result. However, during compression, the second result exhibited a greater number of connections. Nevertheless, the overall trend of increasing connectivity remained similar. This confirms that the Monte Carlo method successfully replicates aspects that should be considered in actual fabrication.

**Figure 3:**
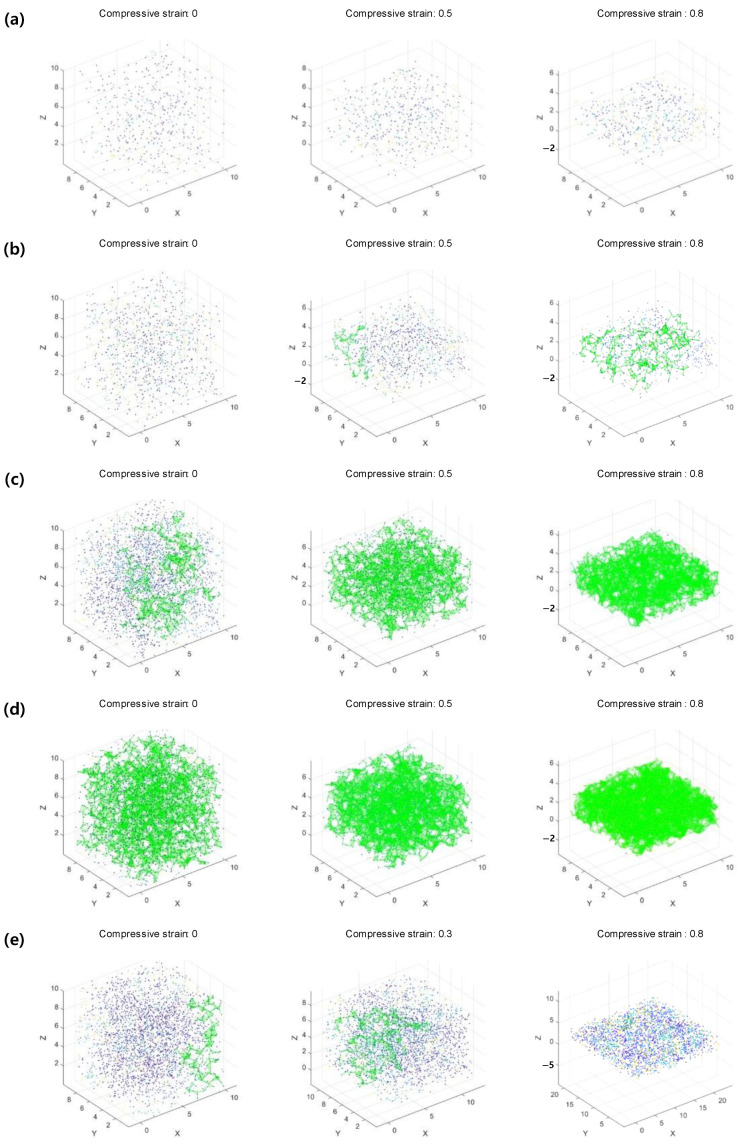

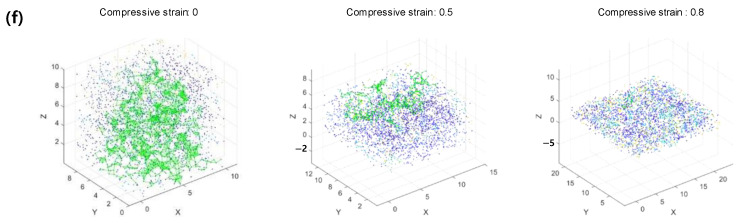
The six cases of the 3D compressive percolation model (**a**–**f**).

The analysis of changes in conductive particle connections, which determine the electrical performance of flexible electronic composites under compressive deformation, revealed six cases using the 3D compressive percolation model. Figure 3 illustrates these six cases of connection changes during compression, with each case representing a characteristic scenario from the overall conditions. Figure 3a represents cases where the number of conductive particles is insufficient both before and after compression, resulting in no infinite cluster formation and, consequently, no electrical conductivity. Figure 3b occurs when the number of conductive particles increases, and while no connections exist before compression, particles connect during compression as inter-particle distances decrease, leading to the formation of an infinite cluster. Figure 3c represents a case where the number of conductive particles is high enough that an infinite cluster exists before compression, and, at a final compressive strain of 0.8, all conductive particles are ideally connected, achieving optimal electrical conductivity. Figure 3d begins with a more extensive infinite cluster, but as all particles connect during compression, the system reaches equilibrium, and no further connections are formed despite continued deformation. Figure 3e is caused by a high Poisson’s ratio in the flexible matrix, where connections initially increase during compression, but expansion in the shear plane increases the distance between particles, leading to the loss of connections and the disappearance of the infinite cluster. Finally, Figure 3f also results from a high Poisson’s ratio, where initially high connectivity decreases during compression, resulting in the disappearance of the infinite cluster. These six cases highlight the diverse effects of compression and particle distribution on the conductive properties of flexible electronic composites.

**Figure 4:**
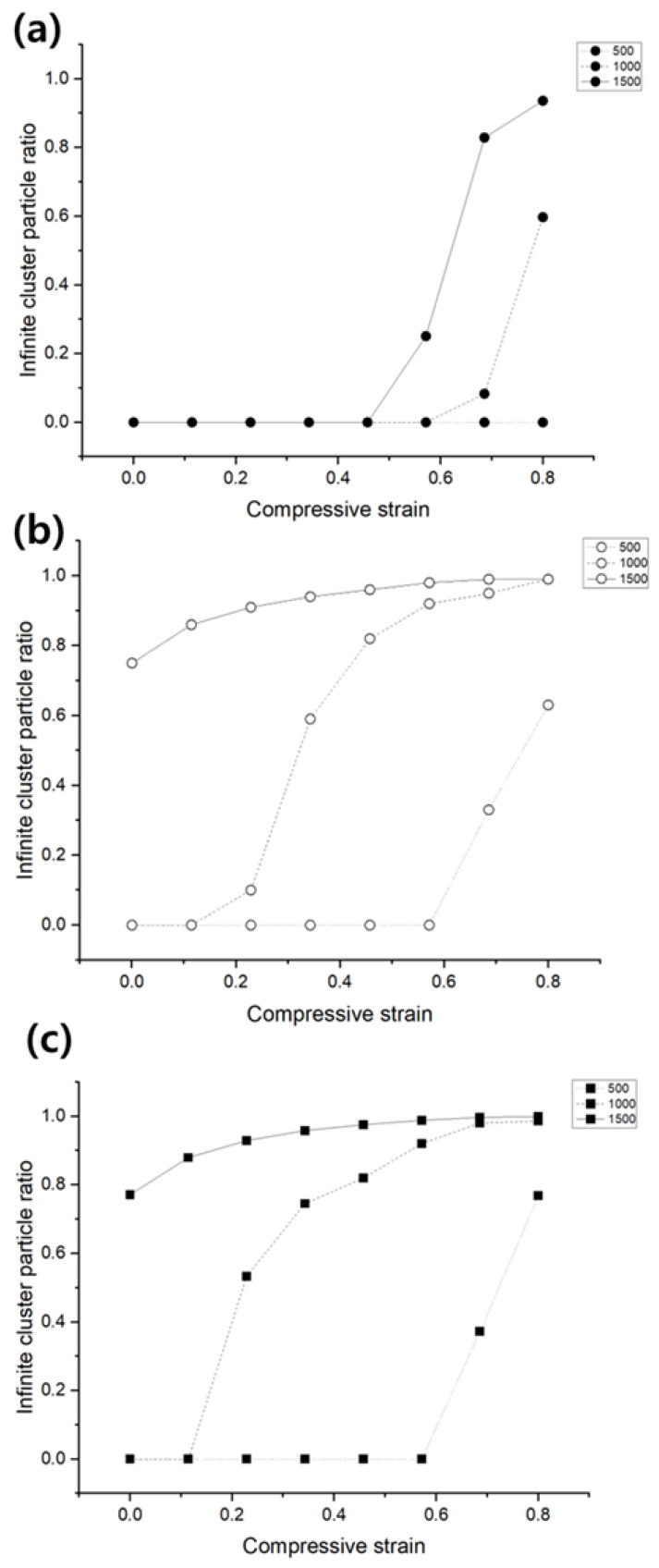
The infinite cluster results in 3 different shapes of conductive particles in 3D compressive percolation model. (**a**) Sphere, (**b**) cylinder, (**c**) rectangular.

Figure 4 illustrates the comparative analysis of finite and infinite cluster formation, and the differences in the number of nodes constituting the clusters, under three representative particle shapes: sphere, cylinder, and rectangular. To isolate the effects of particle shape, all other conditions were kept identical, with particle counts of 500, 1000, and 1500, and the expansion in the shear plane was restricted. In Figure 4a, which shows the results for sphere-shaped particles, infinite clusters did not form under compressive strains up to 0.4. For particle counts of 1000 and 1500, infinite clusters began to form at compressive strains exceeding 0.5 and 0.4, respectively. Figure 4b,c present the results for cylinder and rectangular particles under the same particle count and random volume distribution conditions. For the cylinders with a diameter-to-length ratio of 5 and rectangular particles with a major axis-to-minor axis ratio of 1.5 and a thickness of 0.2, infinite clusters formed more rapidly, with more particles connecting under compressive deformation.

## 4. Discussion

This study leveraged a Monte Carlo simulation approach to explore the behavior of conductive particles in flexible electronic composites under compressive deformation. Variables that are challenging to control experimentally—such as the number and shape of conductive particles, their spatial distribution, and the mechanical properties of the matrix—were systematically analyzed. By generating random distributions for particle positions, sizes, and orientations, the simulation replicated real-world irregularities, enabling robust statistical analysis. These results provided critical insights into the electrical property changes in flexible electronic composites and allowed the classification of deformation behaviors into six cases.

Among the six identified cases, the configuration with the largest variation in the number of connected particles from the initial state to the final compression was found to be the most suitable for achieving high sensitivity. This behavior allows the sensor to detect subtle changes in external forces, making it ideal for applications requiring precise measurement of compressive strain. However, many pressure sensors experience a decline in sensitivity within their operational range due to connection saturation. When the initial number of conductive particles is excessively high, overconnectivity occurs at the start of deformation, limiting the potential for additional connections as compression progresses. This saturation reduces the overall sensitivity of the composite, particularly in scenarios requiring nuanced detection across a broad range of compressive forces.

This study also revealed the influence of matrix properties, particularly Poisson’s ratio, on the behavior of conductive composites. In materials with a high Poisson’s ratio, compressive deformation induces expansion in the shear plane, increasing the distance between conductive particles. This phenomenon leads to the loss of connectivity, resulting in a decline in electrical properties. Such behavior can be analogously understood through the lens of tensile sensors, where stretching increases particle separation and electrical resistance. This finding underscores the need to carefully consider Poisson’s ratio when designing flexible electronic composites for both compression- and tension-based applications.

Another significant aspect of this research involved analyzing the effect of particle geometry on cluster formation and connectivity. Conductive particles were categorized into three representative shapes—sphere, cylinder, and rectangular. By isolating particle geometry as a variable while keeping other parameters constant, this study demonstrated that asymmetry and directional randomness significantly enhance connectivity. Spherical particles, due to their symmetric shape, require higher compressive strain to achieve sufficient connectivity. In contrast, asymmetric shapes such as cylinders and rectangles form connections more readily under similar conditions. For cylindrical particles with a diameter-to-length ratio of 5 and rectangular particles with a major axis-to-minor axis ratio of 1.5, infinite clusters formed more rapidly, with more particles participating in connections during compression. This observation suggests that asymmetric and directionally random shapes are inherently more advantageous for forming robust conductive networks, making them preferable for applications requiring high electrical performance under deformation.

Overall, the findings in this 3D compressive model have direct implications for the design of wearable sensors. Effective sensitivity in these sensors depends on the ability of the material’s electrical properties to respond proportionally to deformation. The results highlight the importance of optimizing the number and shape of conductive particles, as well as the mechanical properties of the matrix, to balance initial connectivity and sensitivity under operational conditions. Materials exhibiting excessive initial connectivity may suffer from saturation effects, reducing their performance in dynamic environments. Additionally, the phenomenon of shear plane expansion due to high Poisson’s ratios provides a critical consideration for material design. For applications involving tensile deformation, strategies to mitigate the loss of connectivity must be employed, such as modifying the matrix material or adjusting particle geometry to enhance resilience against separation.

Future research will focus on incorporating a broader range of variables into the model to maximize sensor sensitivity within the desired sensing range. Examples include factors such as the agglomeration of conductive particles due to van der Waals forces, entanglement effects, particle rearrangement under repeated compression, and the inclusion of partial space matrices, such as fiber fabrics. These additional variables will enable more comprehensive simulations of realistic scenarios. Furthermore, to enhance the analysis, data extracted using the Monte Carlo method in this study will be integrated with artificial intelligence to develop advanced algorithmic models. This approach aims to predict and optimize the behavior of flexible electronic composites under various conditions, providing a more robust framework for sensor design and performance evaluation.

## 5. Conclusions

This study successfully employed a Monte Carlo-based 3D compressive percolation model to analyze the electrical performance of flexible electronic composites under compressive deformation. By simulating variables that are challenging to control experimentally, such as the number, shape, and spatial distribution of conductive particles, the research provided new insights into the behavior of these materials. The findings classified electrical property changes into six distinct cases, emphasizing the importance of factors like initial connectivity, saturation effects, and particle geometry. Additionally, the influence of matrix properties, such as Poisson’s ratio, on conductive particle connectivity was demonstrated, providing a robust framework for optimizing wearable sensor sensitivity and performance.

The expected impact of this study is significant for the development of advanced wearable sensors and flexible electronic applications. For instance, in wearable health monitoring devices, a flexible sensor embedded in a wristband could monitor physiological signals such as heart rate, respiration, or joint movement. By applying the insights gained from this research, the sensor design could be optimized to enhance sensitivity and ensure accurate detection of subtle signals under varying conditions, improving device reliability. In industrial applications, pressure sensors for robotics or soft actuators could be developed using optimized flexible composites. By tailoring particle geometry and connectivity, sensors could achieve consistent performance across a wide range of forces, ensuring accurate and repeatable measurements in dynamic environments. In the field of electronic textiles, integrating these materials into smart fabrics could enable lightweight, flexible clothing that monitors wearer activity and environmental changes. The findings of this study provide a basis for designing textiles with enhanced electrical properties, allowing for efficient signal transmission even under strain.

Overall, the research offers a strong foundation for future advancements in flexible electronic materials, enabling new applications in healthcare, industry, and beyond. By incorporating additional variables and leveraging artificial intelligence for further optimization, the potential of these materials can be fully realized.

## Figures and Tables

**Table 1:** The infinite cluster results from Monte Carlo-based 3D compressive percolation.

Strain	1st Result *	2nd Result *
0	0.28	0.2
0.11	0.62	0.68
0.23	0.84	0.85
0.34	0.93	0.92
0.46	0.96	0.95
0.57	0.98	0.98
0.69	0.99	1.00
0.80	1.00	1.00

* The condition of Monto Carlo is 3000 particles and Poisson’s ratio is 0.

## Data Availability

Data are contained within the article.
